# Detection of stratospheric gravity waves induced by the total solar eclipse of July 2, 2019

**DOI:** 10.1038/s41598-020-75098-2

**Published:** 2020-11-10

**Authors:** Thomas Colligan, Jennifer Fowler, Jaxen Godfrey, Carl Spangrude

**Affiliations:** 1 University of Montana, Missoula, MT USA; 2 Montana Space Grant Consortium, University of Montana, 32 Campus Drive, Missoula, MT 59812 USA; 3 Montana State University, Bozeman, MT USA

**Keywords:** Environmental sciences, Atmospheric science

## Abstract

Atmospheric gravity waves generated by an eclipse were first proposed in 1970. Despite numerous efforts since, there has been no definitive evidence for eclipse generated gravity waves in the lower to middle atmosphere. Measuring wave characteristics produced by a definite forcing event such as an eclipse provides crucial knowledge for developing more accurate physical descriptions of gravity waves. These waves are fundamental to the transport of energy and momentum throughout the atmosphere and their parameterization or simulation in numerical models provides increased accuracy to forecasts. Here, we present the findings from a radiosonde field campaign carried out during the total solar eclipse of July 2, 2019 aimed at detecting eclipse-driven gravity waves in the stratosphere. This eclipse was the source of three stratospheric gravity waves. The first wave (eclipse wave #1) was detected 156 min after totality and the other two waves were detected 53 and 62 min after totality (eclipse waves #2 and #3 respectively) using balloon-borne radiosondes. Our results demonstrate both the importance of field campaign design and the limitations of currently accepted balloon-borne analysis techniques for the detection of stratospheric gravity waves.

## Introduction

The concept for atmospheric gravity waves generated by an eclipse was first proposed in 1970^1^. These waves arise in response to a perturbation in a stably stratified fluid^2^. The nomenclature “gravity wave” arises because gravity is the restoring force, but they are also known as “buoyancy waves”. Gravity waves are fundamental to the transport of energy and momentum throughout the atmosphere, and their parameterization or simulation in numerical models provides increased accuracy to forecasts. Characterizing gravity waves generated by a solar eclipse can lead to improved parameterization and simulation, with an eclipse being a natural “meteorological experiment” where solar radiation is removed for a known amount of time. For 42 years scientists have tried to detect eclipse-induced waves in the lower to middle atmosphere with inconclusive results^3,4^. Here we show the detection of three stratospheric gravity waves with the source being the total solar eclipse of July 2, 2019. Our analysis demonstrates the limitations of currently accepted balloon-borne radiosonde techniques for detection of stratospheric gravity waves. Using two standard analysis methods, wavelet and hodograph, distinct results were found from each method. Two waves from the hodograph method were detected 53 and 62 min after totality and a third wave was found in the wavelet method 156 min after totality.

The August 21, 2017 total solar eclipse over the continental United States marked the first unambiguous detection of eclipse-driven gravity waves in the upper atmosphere^5,6^. However, Space Grant teams conducting an intensive radiosonde campaign in Fort Laramie, WY (ARTSE2017) sought to detect a number of atmospheric processes related to the eclipse, including eclipse-induced gravity waves. The campaign targeted lower flight altitudes, ultimately missing key data in the middle atmosphere crucial to the detection of stratospheric, eclipse-driven waves^7^. Building on the 2017 field campaign, the 2019 field campaign’s focus on gravity wave detection meant flights with higher temporal frequency to higher altitudes. The goal of the 2019 campaign was to measure eclipse-driven waves definitively in the stratosphere. The field campaign profiled the atmosphere for twenty-seven hours surrounding the eclipse by launching Graw DFM-09 radiosondes hourly from twenty-three hours before totality to two hours after with an additional radiosonde launched 30 min prior to totality.

**Figure 1:**
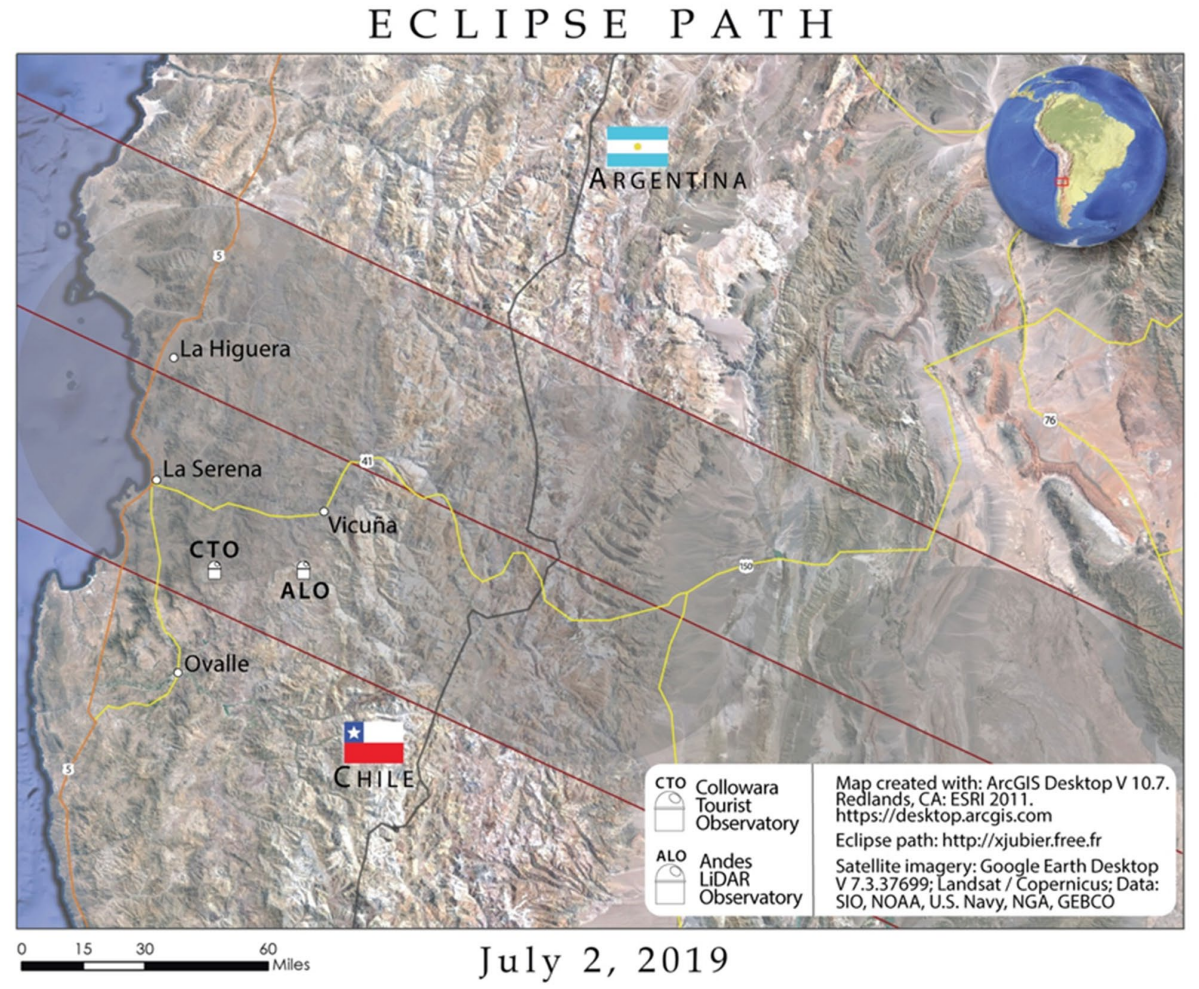
Path of totality for the July 2, 2019 total solar eclipse. The moon’s shadow passed over central Chile and Argentina from the northwest to the southeast. All measurements were made from Collowara Tourist Observatory (CTO) from July 1–3, 2019. Map created by Carl Spangrude using ArcGIS Desktop software ver. 10.7.

The path of totality for the July 2, 2019 total solar eclipse occurred over Chile and Argentina (Fig. 1). All measurements for this field campaign were conducted outside of Andacollo Chile, at the Collowara Tourist Observatory (CTO; − 30.250 S, − 71.063 W). The choice of field site was dictated by the Chilean Directorate General of Civil Aviation and coincident measurements with the Andes LiDAR Observatory (ALO). The field site is a high-elevation (1283 m) desert with an easterly aspect and minimal vegetation. The area immediately surrounding CTO is undeveloped. While CTO was well within the path of totality for the 2019 eclipse, it should be noted that the dynamic terrain of the Andes mountains are themselves a likely source of atmospheric gravity waves. The timing of launches was planned with more launches before the eclipse to characterize the impact of the Andes mountains on the dataset to discern differences from eclipse-induced gravity waves. With the eclipse representing a short-duration event, having three additional hours of data after totality and before sunset represents enough time to see a recovery in the atmospheric state when the eclipse is no longer an influencing event.

Radiosondes are subject to primarily horizontal plane forcing due to wind speed and direction. This reflects the motion of the wind field with altitude and therefore radiosondes effectively detect gravity waves. Gravity waves are elliptically polarized phenomena, and a radiosonde rising through a gravity wave will experience elliptical motion. The elliptical motion is described in the polarization relations for gravity waves and methods of determining gravity wave characteristics rely on calculating elliptical characteristics of wind data. Two methods are commonly used to extract wave characteristics from radiosonde data: the wavelet method and hodograph method^8–11^. The wavelet method performs a wavelet transform and applies Stokes parameters to the transformed data to get elliptical characteristics. The hodograph method fits an ellipse to the zonal and meridional wind data. Applying Stokes parameters directly to the data reveals the same parameters as the hodograph method without additional filtering performed by a wavelet transform. Both methods directly measure intrinsic frequency, horizontal propagation direction, and vertical wavelength. Many other gravity wave parameters, such as horizontal phase speed can be estimated by applying the polarization relations for gravity waves^8^. Eclipse-driven waves are expected to have a range of frequencies, propagation directions similar to the direction of the eclipse (west to east for the 2019 eclipse), horizontal phase velocities greater than zero and vertical wavelengths on the order of hundreds of meters to a few kilometers^4–6^.

Wavelet transformations require that the input data (wind components and temperature) be evenly spaced. To accomplish this, the data was linearly interpolated as radiosondes report data at irregular vertical intervals. The interpolation chosen was based on the resolution of the radiosonde data given an average rise rate of 5 m/s. After interpolation, a third wavelet power surface is formed by summing the u and v wavelet power surfaces in quadrature to incorporate the data from both zonal and meridional components. Wave-like structures that show up in both the zonal and meridional power surfaces will then appear as local maxima in the third power surface^9^. The wavelet transform in the neighborhood of the maxima is inverted and Stokes parameters are calculated. The Stokes parameters allow for the direct calculation of intrinsic frequency and horizontal propagation direction, and other gravity wave parameters can be inferred from the polarization relations. We chose the Morlet wavelet, an amplitude modulated sine wave with approximately five cycles in its envelope^9^. Signals in the data that resemble this wave will project strongly onto the Morlet wavelet and produce local maxima. However, this method is insensitive to wave signatures that do not resemble the Morlet wavelet. To overcome this issue and confirm any additional candidate waves, the hodograph method was also applied to the data^10^.

The hodograph method has been shown to be a strong method for detection of primary waves based on linear theory for a single vertical profile^11^. Hodographs are formed by plotting zonal and meridional wind data on the x and y axes of a graph, respectively. In the presence of a low frequency gravity wave, the hodograph will form an ellipse. Only closed ellipses were considered in this analysis. The least-square fit ellipse’s tilt angle corresponds to the gravity waves’ horizontal propagation direction. The ratio of semi-major to semi-minor axes is proportional to the gravity waves’ intrinsic frequency scaled by the Coriolis frequency. Gravity waves travel parallel or antiparallel to the major axis of the polarization ellipse and the ambiguity in horizontal propagation direction is resolved by calculating the phase shift between the zonal wind and temperature^4^. The vertical wavelength is found from the vertical extent of the ellipse.

Gravity waves can be caused by a multitude of sources, including mountains, wind shear, and frontal systems. To isolate the eclipse as a source, examination of other potential sources was conducted. Mountain waves typically propagate in the opposite direction of the prevailing wind in the intrinsic frame of reference and have ground-based horizontal phase speeds near zero^12,13^. Additional analysis examined wind shear levels in the radiosonde data. Wind shear layers can produce gravity waves with the same average wind speed as the shear layer and with a propagation direction equal to the direction of the change in wind speed^2,14^. In this study, a shear level is characterized by an absolute value change in wind speed of 7.5 m/s or greater within a vertical range of 500 m or less^15^.

## Results

**Figure 2:**
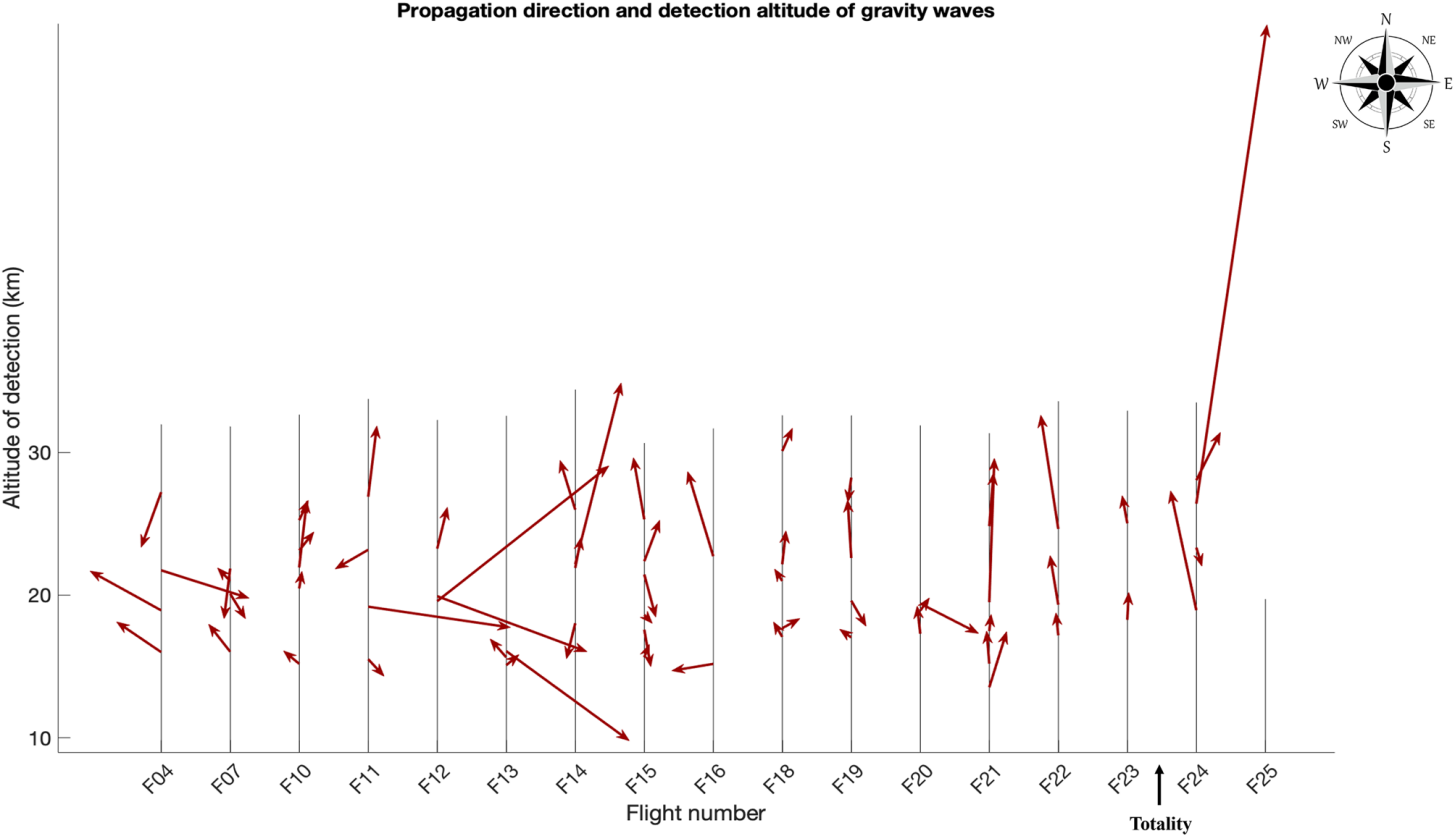
All gravity waves detected by the wavelet method. The red lines point in the direction of propagation of the gravity waves, with north upwards. Flights are labelled on the x-axis, with flights discarded that did not reach at least 12 km. The length of the red line is scaled by the gravity wave’s intrinsic frequency. Clearly, there is a high frequency wave detected in flight #24.

The wavelet-based method revealed one high frequency wave in flight #24 (eclipse wave #1) with an intrinsic period of 40 min, intrinsic frequency, $\hat{\omega }$, here scaled by the Coriolis frequency, $\frac{{\hat{\omega }}}{f}$, of 35, at 26 km in altitude, with a propagation direction of 16°. The horizontal phase speed was 11.5 m/s and vertical wavelength 1.6 km. The ground-based frequency was calculated^8^ and resulted in a value of 0.041 (s^−1^), corresponding to a period of 2.4 min. The mean horizontal phase speed of all waves detected before the eclipse was 4.0 m/s with a standard deviation of 4.3 m/s (n = 52). A mean period of ~ 4 h corresponds to a mean $\frac{{\hat{\omega }}}{f}$ of 5.4 (standard deviation of 5.7). Propagation directions of all waves detected before the eclipse were fairly heterogenous but the majority were traveling between 50–150° (Fig. 2).

**Figure 3:**
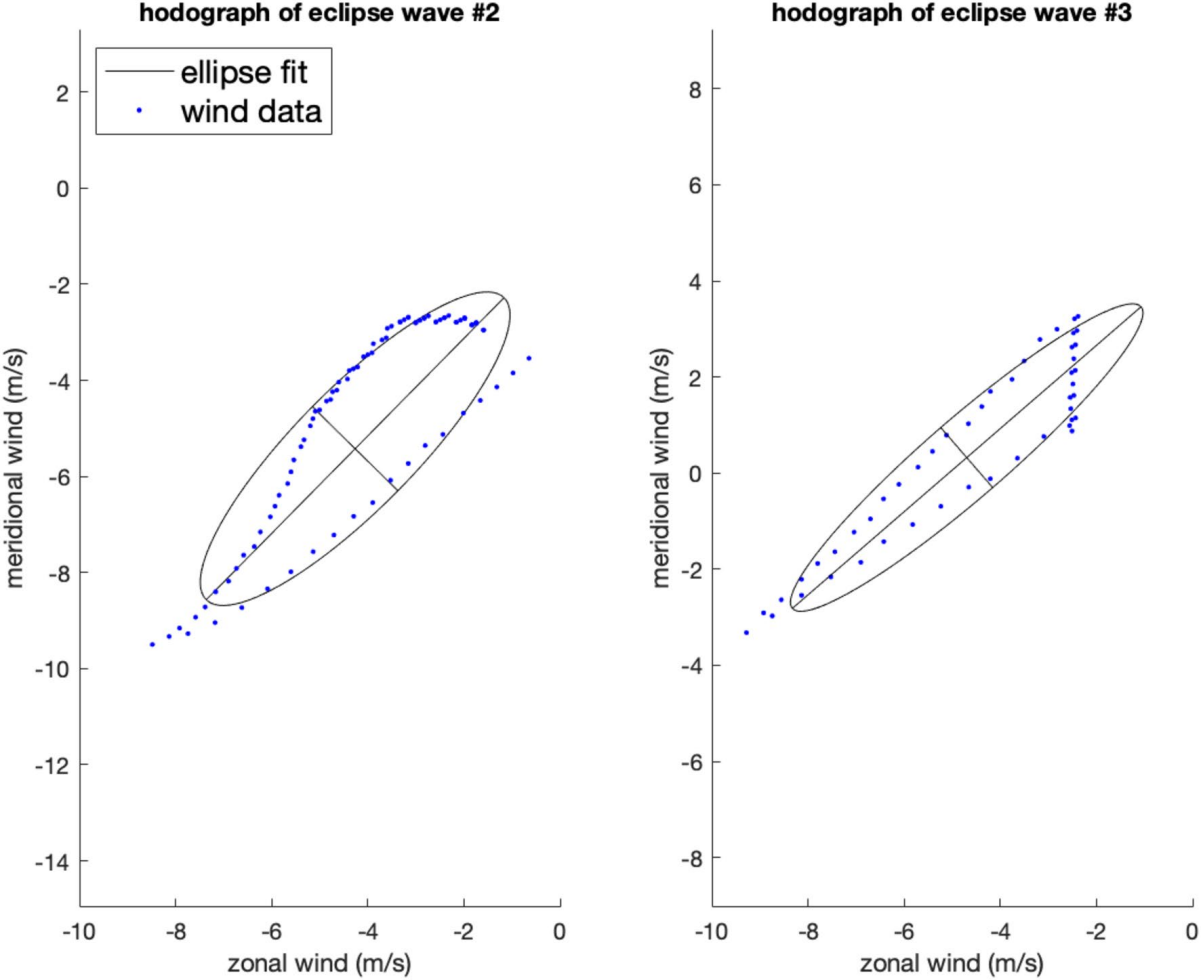
Hodographs for eclipse gravity waves #2 and #3. Negative values on y-axes indicate southerly direction and positive values indicate northerly direction. Negative values on the x-axes indicate westerly values.

**Figure 4:**
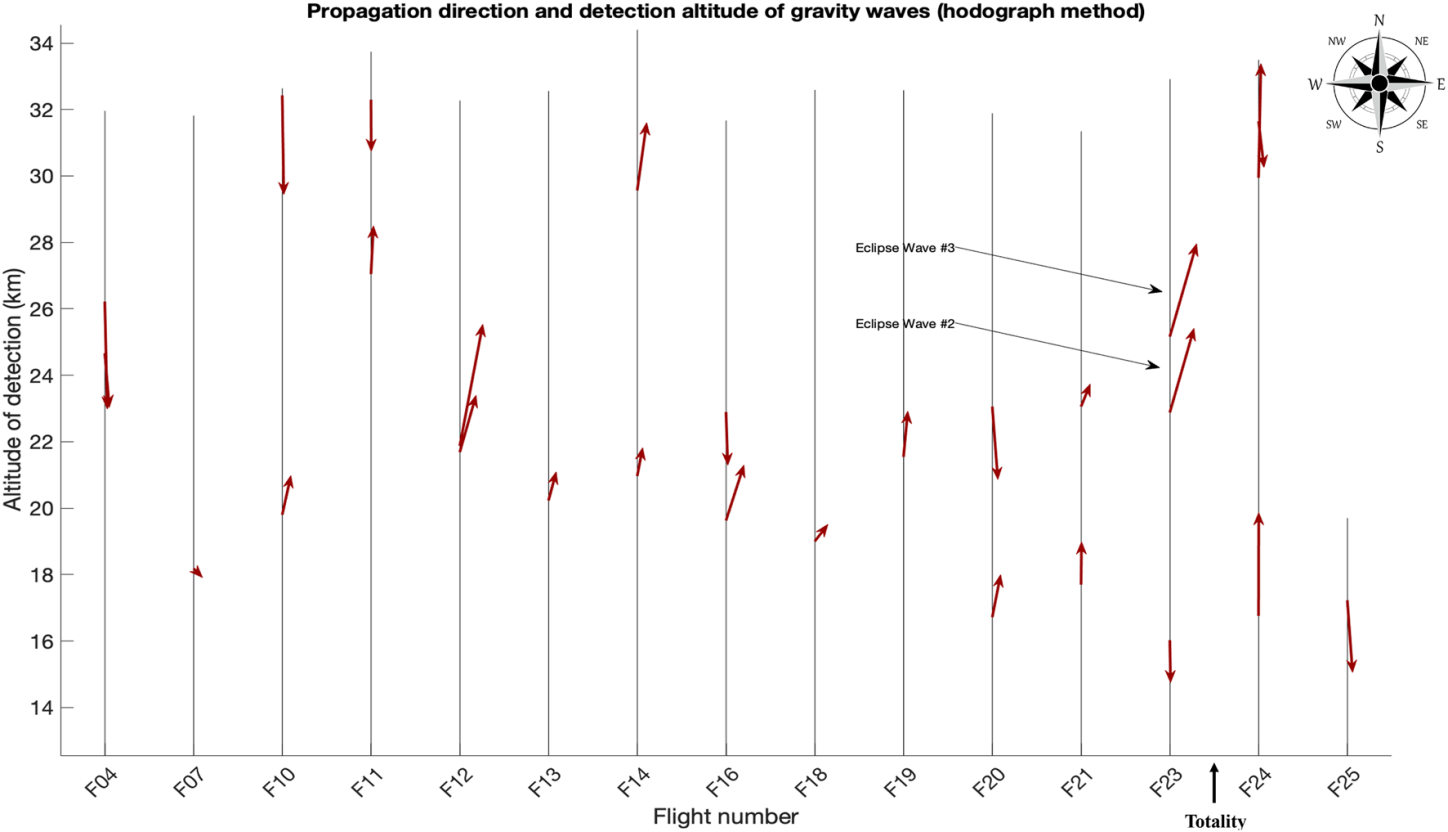
All gravity waves detected by the hodograph method. The red lines point in the direction of propagation of the gravity waves, with north upwards. Flights are labelled on the x-axis, with flights discarded that did not reach at least 12 km. The length of the red line is scaled by the gravity wave’s intrinsic frequency. Waves above 20 km for F23 are eclipse gravity waves #2 and #3. A strong shear level is a probable source of the waves in F12 that is not present in F23.

The hodograph method, which does not apply as rigorous a filtering method inherent in the wavelet method, revealed two waves (eclipse waves #2 and #3) in flight #23 (Fig. 3). Eclipse wave #2 had a period of 6.7 h ($\frac{{\hat{\omega }}}{f}$ of 3.5), was detected at 23 km, and had a propagation direction of 45°. The horizontal phase speed was 4.0 m/s and the vertical wavelength was 400 m. Eclipse wave #3 had a period of 4.0 h ($\frac{{\hat{\omega }}}{f}$ of 5.9), was detected at 25 km, and had a horizontal propagation direction of 49°. The horizontal phase speed was 1.1 m/s and the vertical wavelength 200 m. Both eclipse wave hodographs indicate that the waves were traveling upward, as the direction of rotation of the wind vector is anticlockwise^10^. The mean vertical wavelength of all waves using the hodograph method detected before the eclipse was 2.25 km with a standard deviation of 1.13 km. Mean horizontal phase speed was 19.3 m/s with a standard deviation of 13.5 m/s (n = 18). Mean intrinsic frequency of all waves detected before the eclipse, $\frac{{\hat{\omega }}}{f},$ was 1.9 with a standard deviation of 0.9, corresponding to a mean period of ~ 12 h. Propagation directions of all waves detected before the eclipse were fairly homogenous with the majority traveling between 10 and 180° (Fig. 4).

The hodograph method tends towards uncertainties in the wave parameters from the polynomial fit chosen to separate perturbations from background flow, location of the sounding, and when multiple waves are present^11^. Therefore a Monte Carlo estimation of the parameters was done to arrive at the most probable fit of the ellipse. A visual inspection of the ellipse fits in Fig. 3 may look poor because the points are not evenly spaced and there is a bias in the ellipses shifting towards the northeast possibly due to the transient effect of the eclipse. No data was removed from the hodographs prior to fitting to reduce further bias in the ellipse fits. Along with the preservation of data in the ellipse fits and results from the Monte Carlo estimation of the parameters, there is high confidence in the results indicating the presence of eclipse waves #2 and #3. The wavelet method did not detect eclipse waves #2 and #3 because the oscillation of their signals did not project strongly onto the Morlet wavelet and accordingly did not manifest as peaks in the wavelet power spectrum. Conversely the hodograph method did not detect eclipse wave #1 because it does not form a closed ellipse before application of the wavelet transform.

**Figure 5:**
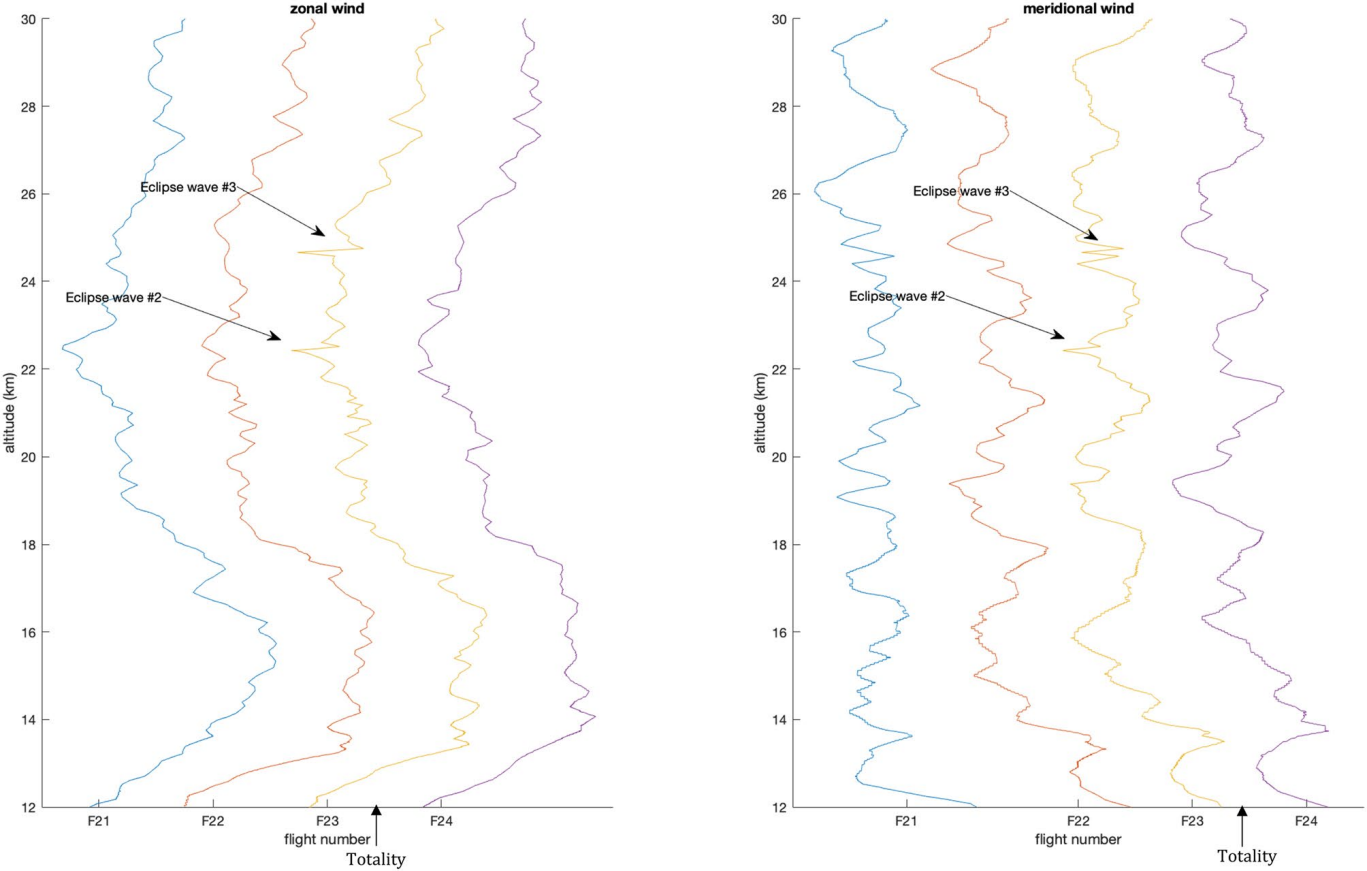
Wind profiles for four soundings (flights F21-24). F21-23 were launched prior to totality and F24 was launched just after totality. Note the shape of the unique perturbations only visible in F23 that are not visible in flights 21, 22, and 24.

The zonal and meridional wind components of flights #21–24 are displayed in (Fig. 5). Flights #21–23 were launched before totality with flight #24 launched after totality. Because of timing, flights #22 and #23 were at altitude during totality with flight #22 approximately 30 min ahead of flight #23 equating to an altitude difference of approximately 9000 m. Most variations in wind data are able to be tracked over time. Distinctive perturbations are visible in flight #23 labelled as eclipse waves #2, #3 in Fig. 5. Eclipse wave #2 occurred 53 min after totality and eclipse wave #3 occurred 62 min after totality in flight #23 while flight #22 had terminated by the time eclipse wave #2 was visible in the data. Wave parameters of eclipse waves #2 and #3 differ from the mean wave parameters detected in other flights. Qualitatively the perturbations associated with these waves appear as Morlet wavelets in both the zonal and meridional wind and are not preceded by another similar signal in flights #21 or #22 shown in Fig. 5. The eclipse wave signals appear and disappear suddenly, with flights #21, #22, and #24 not showing a similar signal. Eclipse waves #1–3 do not have ground-based horizontal phase speeds of zero and were propagating in different directions than expected for mountain waves in the intrinsic frame of reference near the Andes, meaning they were not mountain waves. There were no wind shear levels in the vicinity of eclipse waves #1–3 that could have produced the parameters measured^16,17^. Synoptic conditions were assessed using GOES-EAST satellite data. The atmospheric conditions included relatively little convective activity for the entirety of the field campaign with no frontal system passages. The lack of other sources points to the eclipse as the source of gravity waves #1–3.

## Discussion

To our knowledge the three waves described above represent the first unambiguous detection of eclipse-induced gravity waves in the middle atmosphere. Two factors contribute to this conclusion: (1) the waves have significantly different parameters and signatures when compared to other waves in the data using the same techniques, and (2) other known sources of waves in the atmosphere were not present at the time eclipse waves #1–3 were detected. Suggestions for future campaigns are to repeat this study with consideration of increased flights after the eclipse to replicate this study. These results highlight the large variation in eclipse-driven gravity wave parameters and dissipation characteristics through different atmospheric layers^3–5^. This study advances the field of gravity wave research by providing concrete parameter values for eclipse-driven waves in the middle atmosphere, testing the robustness of both the wavelet and hodograph methods, and providing a template for further successful eclipse studies to try to replicate the findings presented here.

## Methods

### Wavelet transform

Gravity waves are present in a given radiosonde sounding at many different scales and heights. To isolate superimposed gravity waves of different frequencies in a sounding, a wavelet transform can be applied to the collected horizontal wind data using the techniques discussed in Torrence and Compo^18^. There are a variety of wavelet basis functions that can be used in a transform, and common practice is to choose the basis that most closely resembles the signal desired to be detected. Gravity wave signatures in radiosonde profiles are assumed to be amplitude-modulated sine waves based on linear theory, and accordingly we select the Morlet wavelet as a basis function for the transform.

To detect gravity wave packets in the wind data, we use the same method as Zink and Vincent^19^. First the wavelet coefficients of the zonal (*W*_*u*_(*s,z*)) and meridional (*W*_*v*_(*s,z*)) wind components are calculated. The wavelet transform is calculated at a discrete set of scales which must be chosen. Here we choose the set of scales following guidelines in Torrence and Compo^18^, where scale *j* is given by:

1 $$ s_{j} = s_{0} 2^{j\delta j} ,\quad j = 0,1, \ldots ,J $$

2 $$ J = \delta j^{ - 1} log_{2} \left( {\frac{N\delta t}{{s_{0} }}} \right) $$

where *s*_0_ is the smallest resolvable scale, *J* the largest scale, and *N* the length of the time series. *δj* determines the resolution of the transform. The parameters used for the analysis in this paper are *δt* = 1 s, *s*_*0*_ = *2δt*, *δj* = 0.01, and *N* determined the number of points in the profile of interest.

After the wavelet coefficients (*W*_*u*_(*s,z*), *W*_*v*_(*s,z*)) are calculated for all scales of interest, the resulting power surface *S* = |*W*_*u*_(*s, z*)|^2^ +|*W*_*v*_(*s, z*)|^2^ is scanned for local maxima. Values of local maxima less than a threshold value 0.01 m^2^/s^2^ are discarded, as this value corresponds to a perturbation amplitude of 0.1 m/s^19^. Perturbations smaller than this are likely noise. After identifying local maxima, the extension of the corresponding wave packet in scale and height is recorded. The values of *s*_*1*_, *s*_*2*_, *z*_*1*_, and *z*_*2*_ are recorded when the power surface reaches a value of ¼*S* (*s*_*max*_*, z*_*max*_) or starts rising again. After the extent of the packet is recorded, the wind data can be reconstructed using techniques in Torrence and Compo^18^. After the wave packet is reconstructed, the wave parameters must be determined—namely horizontal and vertical propagation directions and intrinsic frequencies. To achieve this we apply Stokes parameter analysis in the same way as Vincent and Fritts^20^.

### Stokes parameter analysis

Stokes parameters were first proposed to estimate gravity wave parameters by Vincent and Fritts in 1987 and give a statistical view on the degree to which the gravity wave field is polarized. The Stokes parameter technique tends to be more accurate than the hodograph method when more than one wave is present in the data or when the analyzed wave is not monochromatic^21^. After the wave packet is reconstructed (resulting in perturbation velocities *u′*, *v′*), Stokes parameter analysis is applied to the data. Stokes parameters were originally developed to describe partially polarized electromagnetic waves, and in analogy we define perturbation velocities *u′* and *v′* as:

3 $$ u^{\prime} = U\left( t \right)cos\left( {\omega t + \delta_{1} t} \right) $$

4 $$ v^{\prime} = V\left( t \right)cos\left( {\omega t + \delta_{2} t} \right) $$

where *U*(*t*) and *V*(*t*) represent the slowly varying amplitudes of the oscillating wave. Given these definitions, Stokes parameters for gravity waves can be defined analogously to those for electromagnetic waves^22^ as:

5 $$ I = \mathop {u^{^{\prime}2} }\limits + \mathop {v^{^{\prime}2} }\limits $$

6 $$ D = \mathop {u^{^{\prime}2} }\limits - \mathop {v^{^{\prime}2} }\limits $$

7 $$ P = \mathop {UVcos\delta }\limits = 2\mathop {u^{\prime}v^{\prime}}\limits $$

8 $$ Q = \mathop {UV^{\prime}sin\delta }\limits $$

In terms of the reconstructed wave packet, *U* = *2u*^*′2*^, *V* = *2v*^*′2*^, and $\mathop {UVcos\delta }\limits$ = $2\mathop {u^{\prime}v^{\prime}}\limits$. Since *u′* and *v′* are assumed to be amplitude-modulated cosine waves, *Q* cannot be calculated directly. However, the reconstructed wave packet contains a real and complex part. The imaginary component represents the 90° phase shifted wind data^19^, allowing for the calculation of *Q*.

Another quantity of interest for this analysis is the “degree of polarization”, *d* = (*D*^*2*^ + *P*^*2*^ + *Q*^*2*^)^*½*^*/I*. Here *d* quantifies the contribution of coherent wave motion to the total velocity variance, with *d* = *0* indicating an entirely incoherent wave field. Wave packets with *d* < 0.5 are discarded as this indicates weak polarization. Results of *d* > 1 are also discarded, as this indicates a deviation of the data from the assumptions in the derivation of Stokes parameters. Additionally, waves with Stokes parameter values of *Q* and *P* < 0.05 are discarded, as this corresponds to weak wave activity^8^.

After computing the Stokes parameters, the horizontal propagation direction and axial ratio (AR) can be calculated using:

9 $$ tan2\theta = \frac{P}{D} $$

10 $$ AR = cot\left( {0.5sin^{ - 1} \left( \frac{Q}{Id} \right)} \right) $$

There is a 180° ambiguity in the horizontal propagation direction that can be resolved using the phase difference between temperature and horizontal perturbation velocities. The sense of rotation of the polarization ellipse is indicated by the sign of *Q*, with a positive value indicating clockwise rotation^23^. For a wave in the southern hemisphere, anticlockwise rotation of the hodograph means the wave is propagating upward.

### Linear theory and the low frequency approximation

Linear theory predicts that the axial ratio of the wind hodograph is proportional to the intrinsic frequency, scaled by the Coriolis frequency at that latitude^23^:

11 $$ AR = \left| {\frac{f}{{\hat{\omega }}}} \right| $$

## Data Availability

The datasets generated and analyzed during the current study are not publicly available due to an agreement with a sponsor but are available from the corresponding author on reasonable request.

## Code availability

The MATLAB algorithms used to analyze and visualize the data presented in this study are available from the corresponding author on reasonable request.
